# The association of XPG and MMS19L polymorphisms response to chemotherapy in osteosarcoma

**DOI:** 10.12669/pjms.295.3747

**Published:** 2013

**Authors:** Yi-lei Zhao, Li-bin Yang, Xiao-lin Geng, Qing-lan Zhou, Hua Qin, Lin Yang, Yu-zhen Dong, Jin-Jie Zhong

**Affiliations:** 1 Yi-lei Zhao, Department of Orthopedic Surgery, the First Affiliated Hospital, Xinxiang Medical College, Xinxiang, China.; 2 Li-bin Yang, Department of Orthopedic Surgery, the First Affiliated Hospital, Xinxiang Medical College, Xinxiang, China.; 3 Xiao-lin Geng, Department of Orthopedic Surgery, the First Affiliated Hospital, Xinxiang Medical College, Xinxiang, China.; 4 Qing-lan Zhou, Department of Orthopedic Surgery, the First Affiliated Hospital, Xinxiang Medical College, Xinxiang, China.; 5 Hua Qin, Department of Orthopedic Surgery, the First Affiliated Hospital, Xinxiang Medical College, Xinxiang, China.; 6 Lin Yang, Department of Orthopedic Surgery, the First Affiliated Hospital, Xinxiang Medical College, Xinxiang, China.; 7 Yu-zhen Dong, Department of Orthopedic Surgery, the First Affiliated Hospital, Xinxiang Medical College, Xinxiang, China.; 8 Jin-Jie Zhong, Department of Histology & Embryology, Xinjiang Medical University, 393 Xinyi Road, Urumqi, China.

**Keywords:** Clinical outcome, MMS19L, Osteosarcoma, XPG

## Abstract

***Objective:*** To assess the role of XPG, XPC, CCNH and MMS19L polymorphisms response to chemotherapy in osteosarcoma, and the clinical outcome of osteosarcoma.

***Methods:*** One hundred and sixty eight osteosarcoma patients who were histologically confirmed were enrolled in our study between January 2007 and March 2009. Genotyping of XPG, XPC, CCNH and MMS19L was performed in a 384-well plate format on the MassARRAY® platform.

***Results:*** Individuals with rs2296147 TT genotype showed a better response as compared with CC genotype, with the OR (95% CI) of 3.89(1.49-10.95). Those carrying rs29001322 TT genotype presented better response to chemotherapy, and the OR (95% CI) was as high as 12.25(2.63-121.84). Patients carrying TT genotype of XPG rs2296147 and MMS19L rs29001322 showed a significantly longer overall survival than CC genotype, they had 0.37 and 0.31-fold risk of death when compared with wide-type of this gene.

***Conclusions:*** XPG rs2296147 and MMS19L rs29001322 are correlated with response to chemotherapy and prognosis of osteosarcoma. Our findings would provide important evidence for prognostic and therapeutic implications in osteosarcoma.

## INTRODUCTION

Osteosarcoma is the most frequent bone tumors, mainly occurring in children and adolescents.^1^ The standard treatment of osteosarcoma is involved neoadjuvant therapy before and after surgery therapy of the primary tumor. Most of the prognostic factors of osteosarcoma included histological response to pre-operative treatment and status of tumor necrosis.^2^ However, the clinical response to chemotherapy is influenced by genetic and environmental factors. Anticancer therapies have a limited therapeutic range, and the high concentration of these anticancer therapies, such as chemotherapy, could causes toxicity, while the low concentration may reduces the efficacy of treatment. Individuals may present differences in response and toxicity of each anticancer drug. Therefore, the genetic factors are involved in the process of influencing the drug absorption, metabolism and excretion as well as distribution, which influence the individual susceptibility to anticancer therapy.

Nucleotide excision repair pathway involved in the DNA repair processes plays an important role in the efficacy of chemotherapy. Previous several studies have shown that the SNPs of NER genes are related with the response to chemotherapy in osteosarcoma.^3^^-^^5^ However, the response to chemotherapy of osteosarcoma by XPG, XPC, CCNH and MMS19L has not been studied. Therefore, in our study, we aimed to assess the role of XPG, XPC, CCNH and MMS19L polymorphisms response to chemotherapy in osteosarcoma.

## METHODS

***Subjects, treatments and clinical variables*** ***: ***One hundred and sixty eight osteosarcoma patients who were histologically confirmed were enrolled between January 2007 and March 2009 in our hospitals. Patients who had secondary or recurrent tumors and a history of other malignant tumor were excluded from our study. All patients were followed up every month by telephone or clinic visiting until death or the end of follow-up, and written informed consents were obtained from all patients.

Patients received intravenous adriamycin at 25 mg/m^2^ at day one for three days and continued for three courses, and 14 g/m^2^ methotrexate plus 35 mg/m^2^ cisplatin at day one and continued for four courses before surgery. While patients received methotrexate 10 g/m^2^, 25 mg/m^2^ cisplatin or adriamycin, 0.45 mg/m^2^, 500 mg/m^2^ cyclophosphamide, and 1.5 mg/m^2^ vincristine at day one for three days and continued for three weeks. All the chemotherapy was repeated for a maximum of six cycles. If patients showed non-hematology toxicity which was higher than grade three, febrile neutropenia or thrombocytopenia with bleeding, the dosage of chemotherapy drug would be reduced by 25%.

***Clinical Assessments: ***The response to chemotherapy was assessed after six weeks of treatment. Patient response to treatment were determined after four cycles by the WHO criteria.^6^ The response to chemotherapy was categorized into good and poor response to chemotherapy. Complete response or partial response were regarded as good response, and stable disease or progressive disease were regarded as poor response. Overall survival (OS) was used to assess the clinical outcome of osteosarcoma, and the OS was assessed from the date of entry to the date of death or last clinical follow-up. All patients were followed up for three years.

***DNA extraction and quantification*** **: **5 ml venous blood was drawn from all patients, and was kept **at** -20ºC. Total DNA was extracted from the buffy-coat layer using a TIANamp blood DNA kit (Tiangen Biotech, Beijing, China) with centrifuging for 3 min at 13.400 x g (12.000 rpm). Genotyping of XPG, XPC, CCNH and MMS19L was performed in a 384-well plate format on the MassARRAY® platform (Sequenom®, San Diego, CA, USA), which combines polymerase chain reaction (PCR) and matrix-assisted laser desorption/ionization time-of-flight (MALDI-TOF) mass spectrometry technologies. PCR and single base extension (SBE) primers were designed using Sequenom® Assay Design 3.1 software (Sequenom®), according to the manufacturer’s instructions. Polymerase chain reaction (PCR) conditions were used as follows: an initial melting step of 5 min at 94 ºC; 35 cycles of denaturation for 30 s at 94°C; annealing for 30 s at 64 ºC; extension for 60s at 72 ºC, followed by a 5 min final extension at 72°C.

***Statistical analysis*** ***: ***The overall survival was defined from the time of patients enrolled to their death regardless of any cause or the end of the study. Association between response to chemotherapy and XPG, XPC, CCNH and MMS19L genotypes was analyzed by logistic regression analysis with odds ratio (OR). Homozygotes for the most frequent allele were regarded as the reference group. Association between genotypes of XPG, XPC, CCNH and MMS19L and overall survival of osteosarcoma was assessed by Cox Hazard regression model with hazard ratios (HR) and their confidence intervals (CI). Survival distributions were estimated by using the Kaplan-Meier method. *P* value less than 0.05 was considered to be significant. All analyses were performed using the Statistical Package for the Social Sciences (SPSS) software 13.0 for windows.

## RESULTS

***Patients*** ***:*** The main clinical and pathological characteristics of one hundred and sixty eight osteosarcoma patients are showed in Table-I. The median age at diagnosis was 16.8(range 6 to 37 years). Almost 85% of the patients were younger than 20 years old at the time of recruitment, and 97 (57.7%) were male. 90.5% of the tumors were located in femur and tibia. At the time of diagnosis, 18.3% of the patients showed metastasis, while 24.6% presented metastasis during the follow up, and 57.1% did not show metastasis. 68.5% of patients showed relapse during the follow-up.

**Table-I:** Clinical and pathological characteristics of included patients

	Patients	
	No	%
Median age, yr (range)	16.8(6-37)	
Sex		
Female	71	42.3
Male	97	57.7
***Location***		
Femur	89	53.2
Tibia/flbula	63	37.3
Arm	10	6.2
Central	6	3.3
***Metastasis ***		
No	96	57.1
At diagnosis	31	18.3
At follow-up	41	24.6
***Histological response***		
Good	94	56.0
Poor	74	44.0
***Death during follow-up***		
Alive	103	61.3
Dead	65	38.7
***Relapse***		
No	115	68.5
Yes	53	31.5

A total of 94 patients presented as good response to chemotherapy, while the remainders (65 patients) were poor responders. The results showed that polymorphisms of XPG rs2296147 and MMS19L rs29001322 affect the response to platinum-based chemotherapy (Table-II). Individuals with rs2296147 TT genotype were more likely to have better response to platinum-based chemotherapy compared with CC genotype, with the OR (95% CI) of 3.89(1.49-10.95). Those carrying rs29001322 TT genotype presented better response to chemotherapy, and the OR (95% CI) was as high as 12.25(2.63-121.84).

**Fig.1:**
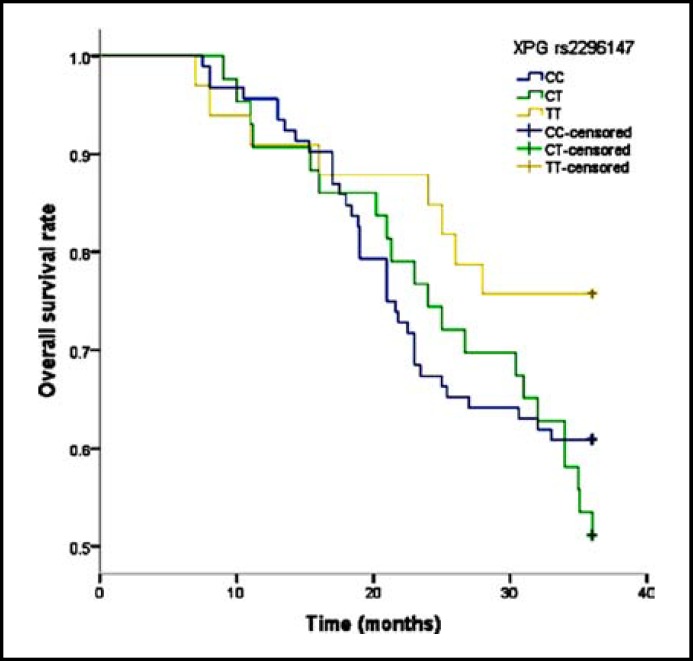
Overall survival of XPG rs2296147 polymorphisms

**Fig.2:**
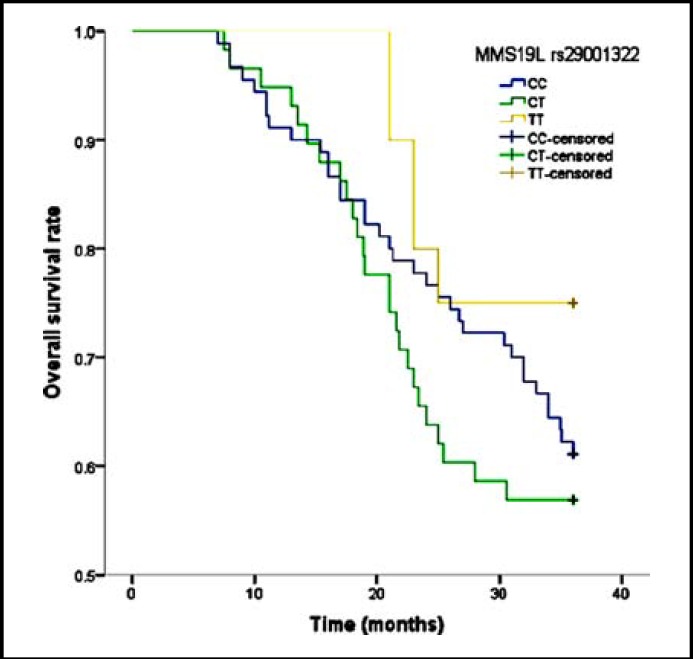
Overall survival of MMS19L rs29001322 polymorphisms

In our study, the median overall survival of patients was 27.5 months. Patients carrying TT genotype of XPG rs2296147 showed a significantly longer overall survival (32.3 months) than CC genotype, they had 0.37-fold risk of death when compared with wide-type of this gene. We found a significantly decreased risk of death from osteosarcoma among patients carrying TT genotype of XPG rs1047768, and HR (95% CI) was 0.32(0.06-0.97) (Fig.1). Moreover, TT genotype of MMS19L rs29001322 was also likely to reduce the risk of death from osteosarcoma when compared with CC genotype, and the HR(95% CI) was 0.31(0.08-0.93) (Fig.2).

**Table-II:** Role of XPG, XPC, CCNH and MMS19L genotypes on response to chemotherapy and overall survival

Genotype		Cases	%	Good responder N=94	%	Odds ratio (95% CI)	P value	Events of deaths N=65	%	Hazard ratio (95% CI)	P value
XPG rs2296147	CC	92	54.74	41	45.2	-	-	36	55.5	-	
	CT	43	25.6	25	27.6	1.73(0.78-3.85)	0.14	21	32.1	1.51(0.68-3.64)	0.18
	TT	33	19.66	25	27.2	3.89(1.49-10.95)	<0.05	8	12.4	0.37(0.15-0.93)	<0.05
XPG rs2094258	AA	70	41.68	35	38.5	-	-	24	36.7	-	
	AG	53	31.55	29	32.1	1.21(0.56-2.63)	0.61	22	33.1	0.71(0.34-1.55)	0.34
	GG	45	26.77	27	29.4	1.54(0.68-3.55)	0.26	20	30.2	0.63(0.25-1.46)	0.25
XPC rs2228001	AA	118	70.23	61	67.2	-	-	47	72.5	-	
	AC	28	16.66	16	17.7	1.24(0.50-3.15)	0.59	10	15.3	0.78(0.31-1.75)	0.44
	CC	22	13.11	14	15.1	1.64(0.59-5.02)	0.31	8	12.2	0.86(0.32-2.35)	0.71
CCNH rs2266690	CC	122	72.65	62	68.3	-	-	49	74.7	-	
	CT	28	16.64	18	19.5	1.74(0.69-4.63)	0.18	10	15.1	0.74(0.31-1.83)	0.46
	TT	18	10.71	11	12.2	1.56(0.53-5.16)	0.42	7	10.2	0.86(0.30-2.62)	0.74
MMS19L rs29001322	CC	80	47.61	34	37.3	-	-	35	54.3	-	
	CT	68	40.47	39	42.8	1.93(0.92-3.96)	0.08	25	38.2	0.68(0.36-1.41)	0.22
	TT	20	11.92	18	19.9	12.25(2.63-121.84)	<0.05	5	7.5	0.31(0.08-0.93)	<0.05

## DISCUSSION

The identification of molecular prognostic and predictive markers could provide important information for customized chemotherapy to improve efficacy of chemotherapy. Previous preclinical and clinical studies have indicated the CXCR4, survivin and MMP9 were associated with the clinical outcome of osteosarcoma.^7^ Cisplatin is one of the effective chemotherapy in treatment of osteosarcoma, while NER pathway plays an important role on removing of DNA adducts induced by platinum compounds**.**^8^ We analyze the association of the response to chemotherapy and clinical outcome of osteosarcoma with polymorphisms in XPG, XPC, CCNH and MMS19L among patients with osteosarcoma.

In our study, we found carrying TT genotypes of XPG rs2296147 and MMS19L rs29001322 conferred an estimated 3.89 and 12.25-fold risk of good response to chemotherapy in osteosarcoma. This result was consistent with results of overall survival, and we found they could reduce 63% and 69% risk of death from cancer.

The association between the XPG polymorphism and response to chemotherapy has been described in various cancers previously.^9^^-^^12^ He et al reported that homozygous of XPG rs751402 increase the chemotherapy response in advanced NSCLC**.**^9^ Italiano et al reported that polymorphisms in XPG was significantly associated with PFS and OS of osteosarcoma, and it could be used for the prediction of clinical response to chemotherapy.^10^ While Liu et al and Sakano et al have suggested that polymorphisms in XPG could potentially be predictive factor for clinical outcome of osteosarcoma cancer.^11^^,^^12^ Only one previous study explore the association between polymorphism in XPG and osteosarcoma risk.^5^ Homozygous of XPG was the reduced efficacy genotype which involved in the DNA repair and replication, and thus influence role of removing of DNA adducts induced by platinum compounds. Our study also has showed variation of XPG is correlated with good response to cisplatin in osteosarcoma.

Our findings have important prognostic and therapeutic implications. Tumors with dysfunctional XPG expression would be predicted to demonstrate sensitivity to cisplatin. XPG is a structure-specific endonuclease, which participates in two incision steps that are critical to the DNA repair process. XPG cleaves the damaged DNA 3’ to the damaged site, nonenzymatically participates in the 5’ incision mediated by the ERCC1 and ERCC4 heterodimer, and stabilizes the DNA repair complex to the damaged DNA. XPG levels are associated with cytotoxicity to cisplatin and iofulven, and potentially to be an important therapeutic target**.**^13^

MMS19 splice variants have specific distinct functional domains, and this gene exerts its function in repairing and transcripting. Specific MMS19 domains a specific role in NER pathway and transcription and contributes to regulating the switch between transcription and NER.^14^ Previous two studies reported that the association between MMS19L and risk of cancer or its prognosis**.**^15^^,^^16^ Our study has showed polymorphism in MMS19 is associated with good response of cisplatin chemotherapy in osteosarcoma, and our study provides evidence for further study to clarify their association.

There were several limitations in our study. Firstly, cases were selected from one hospital, which may not better represent all situations of osteosarcoma cases. Secondly, some other genetic polymorphisms may influence the prognosis of osteosarcoma except for DNA repaired genes. Therefore, further large sample multicenter studies with different ethnicities are warranted to further investigate the DNA repaired polymorphisms on prognosis of osteosarcoma.

In conclusion, XPG rs2296147 and MMS19L rs29001322 are correlated with response to chemotherapy and prognosis of osteosarcoma. Our findings would provide important evidence for prognostic and therapeutic implications in osteosarcoma.
